# Reasons for long-term care need: analyzing combinations of health limitations in Germany

**DOI:** 10.1007/s00391-025-02498-2

**Published:** 2025-09-23

**Authors:** Martin Wetzel, Andrea Cass, Johanna Schütz

**Affiliations:** BZPD—Bavarian Center for Digital Health and Social Care, Kempten University of Applied Sciences, Bahnhofstraße 61, 87435 Kempten, Germany

**Keywords:** Cognitive disorders, Functional limitations, Latent class analysis, Health, Medical service, Kognitive Störungen, Funktionale Einschränkungen, Latente Klassenanalyse, Gesundheit, Medizinischer Dienst

## Abstract

**Background:**

Health limitations affect long-term care (LTC) needs differently. For instance, people with cognitive limitations require more organizational support, whereas those with functional limitations require more personal care. While the impact of singular health limitations on LTC has been widely studied, little attention has been given to the prevalences of co-occurring health limitations that drive LTC needs.

**Objectives:**

Our exploratory study seeks to address the gap in understanding the prevalence of multiple, intertwining health limitations that contribute to the need for LTC.

**Materials and methods:**

We used data from the German Medical Service (MD). The MD assesses LTC needs and assigns care grades, which serve as the basis for LTC insurance benefits. The available data contains all assessments in 2019 of adults living in Bavaria (the largest state in Germany), focusing on those with first-time LTC needs (*N* = 101,227). Using latent class analysis, we identified combinations of limitations across six health dimensions (e.g., mobility, cognition).

**Results:**

Among first-time LTC recipients, 5 distinct classes of care needs were identified. Two classes reflect single limitations: mobility limitations, and the need for assistance with medical therapy. Three classes point to various combinations of limitations. While classes differed in size, they also varied significantly by age, gender, and care grade.

**Conclusion:**

The co-occurrence of health limitations is not an exception but a central feature of LTC needs even at the initial stages of dependency, emphasizing the importance of tailored care strategies. These insights can help local authorities and care providers offer targeted LTC services more strategically.

**Supplementary Information:**

The online version of this article (10.1007/s00391-025-02498-2) contains supplementary material, which is available to authorized users.

## Abstract (de)

**Hintergrund:**

Pflegebedürftigkeit unterscheidet sich je nach zugrundeliegender gesundheitlicher Ursache. Menschen mit kognitiven Einschränkungen benötigen beispielsweise andere Unterstützung als Menschen mit funktionellen Einschränkungen. Während der einzelne Einfluss von gesundheitlichen Einschränkungen auf den Pflegebedarf umfassend untersucht worden ist, wurde den Prävalenzen von zusammen auftretenden Einschränkungen bisher wenig Aufmerksamkeit geschenkt.

**Fragestellung:**

Diese explorative Studie untersucht, welche gesundheitlichen Beeinträchtigungen gleichzeitig zu Beginn einer Pflegebedürftigkeit auftreten.

**Material und Methoden:**

Es wurden Daten des Medizinischen Dienstes (MD) Bayern verwendet. Der MD begutachtet den Pflegebedarf und errechnet Pflegegrade, die als Grundlage für Leistungen der Pflegeversicherung dienen. Die Daten enthalten alle Erstbegutachtungen im Jahr 2019 aus Bayern (*N* = 101.227). Mittels latenter Klassenanalyse wurden Muster von gesundheitlichen Einschränkungen in sechs Gesundheitsdimensionen (z. B. Mobilität, Kognition) analysiert.

**Ergebnisse:**

Bei Pflegebeginn wurden fünf verschiedene Klassen von Pflegebedarf identifiziert. Zwei Klassen spiegeln einzelne Einschränkungen wider: Mobilitätseinschränkungen und der Bedarf an Unterstützung bei therapeutischer Versorgung. Drei Klassen weisen auf verschiedene Kombinationen von Einschränkungen hin. Diese Muster unterschieden sich nach Alter, Geschlecht und Pflegegrad.

**Diskussion:**

Die Studie zeigt, dass die überwiegende Mehrheit von Personen mit beginnendem Pflegebedarf eine Kombination von gesundheitlichen Einschränkungen aufweist. Die Erkenntnisse können helfen, gezieltere Pflegeleistungen anzubieten.

## Introduction and background

In the coming years, the number of people in long-term care (LTC) in Germany is expected to rise considerably as the population ages [27]. Different health impairments result in varying care needs, for instance, cognitive limitations require psychiatric care while mobility limitations require physical care. Moreover, individuals may have multiple co-occurring limitations. This complexity underscores the importance of moving beyond mean-level prevalence statistics and instead identifying subgroups of individuals characterized by particular combinations of impairments. Identifying these patterns is vital for ensuring appropriate services are provided to effectively address individual care needs as well as for the planning and allocation of healthcare resources.

In this study, reasons for LTC are defined as the specific combinations of health limitations that impact autonomous living, in accordance with the German LTC insurance system’s framework. Eligibility for LTC support is determined through a standardized assessment conducted by an independent evaluator, such as the German Medical Service (MD). This process examines the level of independence across six dimensions of competence (detailed in section A1 of the online appendix), which contribute unequally to the overall care score, reflecting the degree to which individuals require support:

1. *Mobility (10%): *the ability to move primarily within but also outside the home.
2. Cognitive and communicative abilities (15%*): the ability to communicate and interact, remember, and orientate oneself.
3. Mental issues and behavioral patterns (15%*): the ability to cope behaviorally with everyday problems and the appearance of psychological issues.
4. *Self-care (40%): *the ability to manage personal care tasks like eating and dressing.
5. *Coping with disease- or therapy-related demands (20%): *the ability to manage medication and therapy as well as adapt to the consequences of illnesses.
6. *Managing daily life and maintaining social contacts (15%): *the ability to organize daily activities and maintain social relationships.

*Regarding items 2 and 3, only the dimension with the highest value is awarded 15%.

Dependence in any dimension is interpreted as a health limitation. Based on a weighted sum score, individuals are assigned either a ‘care grade’ (ranging from 1 to 5), which indicate the severity of need and correspond to different levels of LTC insurance benefits, or the request is neglected.

Our analysis focuses on first-time assessments of care needs, providing key insights into the onset of LTC trajectories. Much of the existing research focuses on health limitations in isolation, without fully addressing the complexity of cases in which multiple factors coexist. While several studies have examined the associations between specific limitations—or the interconnection between two limitations—and LTC needs, there remains a gap in understanding the prevalence of multiple, intertwining impairments that contribute to the need for care. Our exploratory study seeks to address this gap by answering three research questions:

- Q1: Which typical combinations of health limitations exist and how are they associated with LTC in older adults?
- Q2: What are the prevalences of these combinations at the time of first assessment?
- Q3: How strongly do the combinations vary by gender, age, or care grade?

### State of research

Research on the health-related reasons for LTC is ample, yet most studies have focused on the link between separate specific limitations and LTC. Cognitive impairment, particularly dementia, is cited as a leading reason for care [3, 14]. Functional impairment is also a key determinant of LTC dependency, as most individuals requiring LTC experience limitations in performing activities of daily living (ADLs), such as bathing and grooming, and mobility-related activities, such as transferring and walking [4, 14]. Challenges in these areas hinder independence and increase the likelihood of needing LTC [14]. Mental health conditions such as depression are also common among LTC recipients [19], with depression prevalence among recipients in Germany estimated to be around 30% [7, 16, 23].

Although much of the existing research has focused on singular impairments in isolation, some exceptions have explored the interplay between multiple conditions, such as the overlap between dementia, functional decline, and mental health issues. Some studies suggest between 70 and 80% of individuals with dementia receiving LTC also encounter a deterioration in their functional health [22, 29]. LTC recipients with dementia may also face mental health issues such as depression [15]. Moreover, mental health conditions have been shown to follow functional decline [20]. In instances where functional limitations are accompanied by dementia, depression rates have been shown to increase with the number of functional impairments [13].

### Age, gender, and degree of care need

Reasons for LTC may vary by age and gender. Health impairments and corresponding LTC needs tend to occur more often in later life [3, 27]. Additionally, men and women age differently and experience different LTC needs. Men often rely on their female spouses for care [21], while women—who generally outlive their male counterparts but tend to experience more health issues, especially functional limitations [1, 2, 8]—are at a heightened risk of requiring formal care as they age [5, 18]. Women are also more likely to receive a care grade [8] and face increased intensity of care need in later life [11]. However, upon initial assessment, the proportion of individuals with a care grade of 3 or higher tends to be larger for men compared to women [15, 24].

At all care grades, difficulty with self-care is highly prevalent, with mobility impairments also being common. The proportion of individuals experiencing mobility impairments tends to rise with higher care grades. Cognitive and communicate impairments are more frequently observed among those with higher care grades as well [24]. In particular, dementia has been found to be the most common initial diagnosis among those with care grades 3 and above [3].

## Study design and investigation methods

### Data

We used data from the 2019 assessments of the German Medical Service (MD) in Bavaria, the largest federal state in Germany. This dataset contains information deduced in the initial assessments determining eligibility for LTC insurance benefits from the year 2019. Based on this, individuals received a ‘care grade’ that forms the basis for cash and in-kind benefits. Access to the data was exclusively granted to Bavarian Center for Digital Health and Social Care (BZPD), University of Applied Sciences Kempten. For a more detailed introduction to the data and its potentials, see [24].

For the current study, our analysis focuses on first-time care assessments for individuals who received a care grade. We do not include cases where a care grade was not assigned (*N* = 25,404), as these individuals did not meet the criteria for LTC needs under German LTC legislation. Those who did not receive a care grade were more often female (4.7% higher probability than men) and younger (0.5% lower probability by year) [q.v., 25]. The final sample included 101,227 individuals who were awarded a care grade. For all individuals, no missing data was present.

### Method

We used latent class analysis (LCA) to identify distinct patterns of limitations that underpin the assessed need for LTC. This approach assumes that the population is heterogeneous and can be divided into mutually exclusive and exhaustive latent classes. Each latent class represents a group of individuals who share similar characteristics or response patterns, though these groups are not directly observed in the data [10]. Based on the patterns of limitations, the probabilities of being a member in a latent class are estimated for each person (class membership probabilities). Moreover, LCAs provide the likelihood of a specific response in an observed variable for each latent class (item-response probability). While LCAs can handle categorical observed variables, it is customary [e.g., 12, 28] to dichotomize these variables to reduce the complexity of the underlying matrices for the LCA. Thus, we categorized each dimension of competence as either *almost fully independent* (1) or *at least slightly in need of assistance* (2). Replication files can be found at https://osf.io/pcj42/.

To identify to the optimal number of classes, no clear criteria are available. Following previous LCA research, we estimated models with a stepwise increase in the number of classes and compared their relative model fit criteria (AIC, BIC, aBIC) and entropy. Lower values of these criteria indicate better fit, with the optimal number of classes being one that balances fit with parsimony [6]. While these criteria often point to multiple viable solutions, taking the interpretability of the classes and some theoretical considerations into account is crucial for model selection. We applied the poLCA-package (version 1.6.0.1) in *R* (version 4.2.3) [17].

To describe key characteristics of each class, we estimated linear regression models for gender (0:men/1:women), age (linear) and care grade (linear) separately using class (dichotomized) to indicate different proportions (for gender) or means (for age and care grade) in the dependent variable (see Online Appendix Tab. A2 for full models). Average margins were predicted for age, gender and care grade, providing mean levels for each variable within each class, along with confidence intervals to indicate class differences.

## Results

Table 1 reveals that among first-time LTC seekers, 66% exhibit at least a slight need of assistance in mobility, 56% in communicative and cognitive abilities, 15% in mental health, 53% in self-care, 78% in self-therapy/-medication, and 81% in daily activities.

**Table 1:** Prevalences of reasons for long-term care need in percent of full sample

		Men	Women	Total
		*N* *=* *39,966*	*N* *=* *61,261*	*N* *=* *101,227*
1	Mobility	64.51	66.19	65.53
2	Communicative/Cognitive	58.53	53.52	55.50
3	Mental health	15.90	14.03	14.77
4	Self-care	56.96	49.87	52.67
5	Self-therapy	80.94	76.64	78.34
6	Daily life	84.78	78.83	81.18

Table 2 presents model fit indicators for successively complex models with increasing numbers of latent classes. These indicators (BIC, aBIC, cAIC, and *χ*^2^) suggest model improvement with each additional class, though the degree of improvement diminished with added complexity. Based on entropy scores, which reflect greater within-class similarity at higher values, we considered a 4- or 5‑class solution optimal. After plotting item–response probabilities for both models, the 5‑class solution displayed a clearer, more interpretable pattern of limitations, and was selected as the final model.

**Table 2:** Model succession

Model	Log-likelihood	Df	BIC	aBIC	cAIC	Entropy	*χ*^2^	Diff. χ^2^	Diff. df	*p* value
Model 1	−349,014.3	57	698,097.7	698,078.7	698,103.7	–	69,364	–	*–*	–
Model 2	−330,751.4	50	661,652.5	661,611.2	661,665.5	0.674	32,839	−36,526	−7	0.00
Model 3	−319,988.3	43	640,207.2	640,143.6	640,227.2	0.743	11,313	−21,526	−7	0.00
Model 4	−318,415.8	36	637,142.7	637,056.9	637,169.7	0.794	8167	−3145	−7	0.00
Model 5	−316,726.4	29	633,844.6	633,736.5	633,878.6	0.784	4789	−3379	−7	0.00
Model 6	−315,703.9	22	631,880.3	631,750.0	631,921.3	0.744	2744	−2045	−7	0.00

**Fig. 1:**
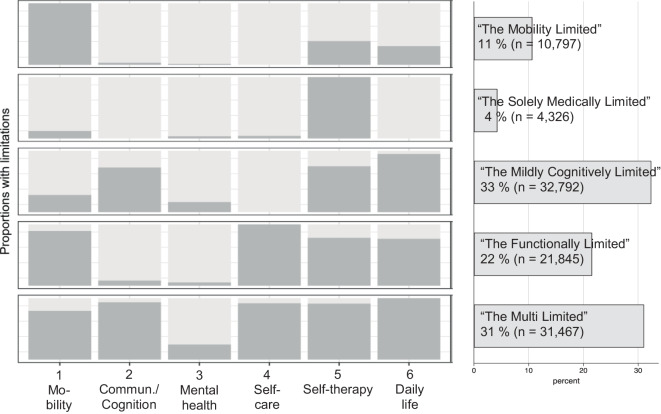
Response probabilities for 5 classes and respective prevalences. *Commun*. Communicative

Figure 1 displays item–response probabilities for each competence dimension in the 5‑class solution. These probabilities depict the likelihood of individuals in each class having limitations in specific dimensions, thereby reflecting reasons for LTC. *Class 1*, comprising 11% of the sample, is characterized by a very high probability of mobility limitations, with low to average probabilities in other dimensions; we labeled this class ‘The Mobility Limited’. *Class 2*, at 4%, is the smallest and includes individuals needing support primarily with self-therapy, hence labeled ‘The Solely Medically Limited’. *Class 3*, the largest, encompassing 33% of the sample, shows high probabilities of impairment in daily life activities, self-therapy, and communication and cognition, and was labeled ‘The Mildly Cognitive Limited’. *Class 4* (22%) has high probabilities of limitations in mobility, self-care, self-therapy, and daily life but not in communication and cognition or mental health. We refer to this class as ‘The Functionally Limited’. Finally, *Class 5*, the second-largest (31%), has very high probabilities across all dimensions, acquiring the label ‘The Multi Limited’. Although mental illness is generally rare in the sample, Class 5 exhibits the highest likelihood.

**Fig. 2:**
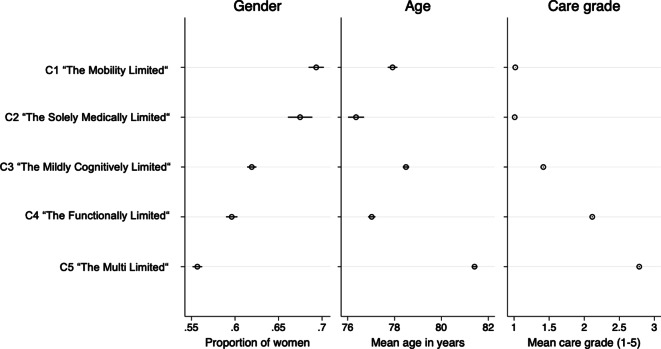
Predicted mean levels (with confidence intervals) for key characteristics of the latent classes

Predicted means for key characteristics of the 5 classes are shown in Fig. 2. Confidence intervals derived from bivariate regression models predicting gender, age, and care grade highlight significant differences across classes. *Class 1*, ‘The Mobility Limited’, predominantly includes women, with an average age and a mean care grade close to 1. *Class 2*, ‘The Solely Medically Limited, also consists of more women than men and members with low care grades. This group is the youngest, with an average age of 76.4 years. *Class 3*, ‘The Mildly Cognitively Limited’, represents individuals of average age and gender distribution, with a mean care grade of 1.4. *Class 4*, ‘The Functionally Limited’, and *Class 5*, ‘The Multi Limited’, are by tendency more male. Members of *Class 4* are younger but have higher care grades, while *Class 5* contains the oldest individuals and has the highest mean care grade.

## Discussion and practical recommendations

This paper explores whether there are typical combinations of limitations that underlie the reasons for LTC among first-time assessed individuals in Bavaria with public health insurance. We identified five distinct patterns of limitations, representing different reasons for LTC. Two classes reflect limitations in singular dimensions—mobility (*Class 1*) and self-therapy (*Class 2*). The remaining three (*Classes 3, 4*, and *5*), and most prevalent, reveal reasons for LTC involving co-occurring limitations. The combination of limitations may create complexity in support demands.

In line with previous research [3, 14], our findings suggest that cognitive and communicative limitations as well as functional impairments are the most common reasons for LTC, with a substantial portion of individuals exhibiting both simultaneously. In contrast, we found that mental health limitations were less prevalent than expected among German LTC recipients [7, 16, 23] and were mostly clustered in “The Multi-Limited” class. While this might indicate that few people have mental health needs, it could also reflect that the German care assessment tool does not consider them dependent enough to receive care benefits. More broadly speaking, the German care assessment is built on an underlying weighting of health limitations and reflects just one possible way of measuring care need.

Regarding severity, we find that individuals with singular limitations typically have lower care grades than those with multiple limitations. Higher care grades translate to more LTC insurance benefits, allowing for more professional care support.

Our findings also highlight gender differences: women predominate in classes with singular limitations. *Classes *with multiple limitations, though majority female, include a higher proportion of males and correspondingly higher care grades. This may be due to men from these cohorts (mostly born between 1929 and 1956) being less accustomed to self-care tasks such as meal preparation and laundry, or to men having fewer but more severe functional limitations.

The current analysis suggests that those with single impairments may experience a more gradual need progression, whereas those with multiple limitations likely apply for LTC benefits later, with higher severity, possibly due to acute health events. Future research should apply a longitudinal perspective and include the family support network. Furthermore, it would be a unique opportunity to explore whether individuals enter LTC gradually or upon health shocks, using large-scale routine insurance data. Based on the current findings, we would expect that about 4% (here, The Solely Medically Limited) enter with a severe health event, but an additional 31% (here, The Multi-Limited) enter LTC either due to health shocks or because a partner (mostly the wife [9]) has compensated for the first occurrences of LTC needs. Accordingly, it would be valuable to examine family structure differences between these groups. Additionally, previous research has linked cognitive impairment with earlier transitions to institutional care [26], but without accounting for other reasons for LTC, it remains uncertain whether these transitions are driven by cognitive impairment alone or the co-occurrence with other limitations.

This study has several strengths and limitations. We analyzed a comprehensive regional population of publicly insured, first-time assessed LTC recipients. Focusing on initial assessment reduces the potential heterogeneity in reasons for care and may mean that the prevalences of classes with more severe limitations are underestimated, or that additional classes might emerge if the full population of care receivers would be addressed. Moreover, this analysis does not fully represent the population with early LTC needs in Bavaria, as it excludes certain individuals: not included in the data are those with private health insurance who are assessed by another agency, namely MedicProof (e.g., civil servants, self-employed individuals), and those who do not undergo an assessment. This may be due to sufficient financial means to cover LTC privately or to personal reasons, such as shame associated with care benefits. Moreover, the data lack additional sociodemographic variables that could help answer the research question at hand. This is because the primary purpose of the data collection is to determine eligibility for care grades, not to support scientific research.

## Conclusion

This analysis offers novel insights into the different combinations of health limitations at the onset of one’s long-term care (LTC) biography. Among first-time LTC recipients, three equally prevalent patterns of need emerged: primarily mild cognitive limitations, primarily functional limitations, and limitations in nearly all dimensions. A key finding is that co-occurring limitations are common, affecting the vast majority of those with first-time LTC benefits. These individuals present with more severe care needs and are, by tendency, more often male compared to other groups.

Several practical implications can be drawn out of our study: for care providers, these findings underscore the importance of addressing multiple limitations. In particular, in situations where care is provided to support functional limitations, programs for preventing cognitive decline may be complementary. This complexity calls for a highly trained workforce to ensure adequate care delivery.

Local governments and communities—particularly those developing age-friendly strategies—should design services that address physical and cognitive impairments simultaneously. Dementia-friendly infrastructure and inclusive communication strategies should go hand-in-hand with physical accessibility.

Co-occurring limitations can create challenges for individuals and families. Given the prevalence of multiple limitations already at the first assessment, support strategies should be expanded to strengthen and stabilize these arrangements and reduce informal caregiver burden. Integration of consultation services addressing both functional and cognitive needs is preferable over domain-specific consultation services.

From a broader perspective, these findings suggest that LTC systems already face high-intensity care needs at the moment of initial assessment. These cases with more complex needs weigh financially on the system. Investing in prevention, community-based care, and caregiver support can help mitigate strain.

## Supplementary Information

Overview of the assessment instrument in English and some further statistics

## Declarations

This article does not contain any studies with human participants or animals performed by any of the authors.
