# Supplementary material for: Reasons for long-term care need: analyzing combinations of health limitations in Germany
Source: Z Gerontol Geriatr. 2025 Sep 23;59(2):125–31. doi: 10.1007/s00391-025-02498-2 (PMC12953248; doi:10.1007/s00391-025-02498-2)
Supplement: Supplementary file 1 — Overview of the assessment instrument in English and some further statistics [file 391_2025_2498_MOESM1_ESM.pdf]

Online Supplement to

M. Wetzel, A. Cass and J. Schütz

Reasons for long-term care need: Analyzing combinations of health limitations in Germany

Ursachen von Pflegebedarf:

Eine Analyse der Kombinationen von gesundheitlichen Beeinträchtigungen in Deutschland

Zeitschrift für Gerontologie und Geriatrie

https://doi.org/10.1007/s00391-025-02498-2

A1. Overview of the Assessment Instrument – English version

The following are own translations.

Mobility	
Changing position in bed<br>Maintaining a stable sitting position<br>Transferring<br>Moving within the living space<br>Climbing stairs	Independent
	Mostly independent
	Mostly dependent
	Dependent
	Mostly independent
Special needs constellation: inability to use both arms and legs	No
	Yes

<table>
<tr><th colspan="2">Cognitive and communicative abilities</th></tr>
<tr><td rowspan="4">Recognition of people in the immediate vicinity
Spatial orientation
Temporal orientation
Recalling essential events or observations
Managing multi-step daily activities
Making decisions in daily life
Understanding facts and information
Recognizing risks and dangers
Communicating basic needs
Understanding requests
Participating in a conversation</td><td>Present/unaffected</td></tr>
<tr><td>Largely present</td></tr>
<tr><td>Present to a small extent</td></tr>
<tr><td>Not present</td></tr>
</table>

<table>
<tr><th colspan="2">Behavioral and psychological problems</th></tr>
<tr><td rowspan="2">Motor-related behavioral abnormalities
Nighttime restlessness
Self-harming and self-aggressive behavior
Damaging objects
Physically aggressive behavior towards others
Verbal aggression
Other care-relevant vocal disturbances</td><td>Never or very rarely</td></tr>
<tr><td>Rarely</td></tr>
</table>

Refusal of care and other supportive measures<br>Delusions<br>Anxieties<br>Loss of motivation in a depressive state<br>Socially inappropriate behaviors<br>Other care-relevant inappropriate behaviors	Frequently<br><br>Daily

Self-care	
Washing the front upper body<br>Body care in the head area<br>Washing the intimate area<br>Showering and bathing including hair<br>Dressing and undressing the upper body<br>Dressing and undressing the lower body<br>Preparing food in bite-sized portions and pouring drinks<br>Eating<br>Drinking<br>Using a toilet or commode<br>Coping with the consequences of urinary incontinence and	Independent<br><br>Mostly independent<br><br>Mostly dependent

dealing with permanent catheters and urostomies<br><br>Coping with the consequences of fecal incontinence and dealing with a stoma	Dependent
Parenteral or tube feeding	Provided independently
	Non-daily, non-permanent assistance
	Daily assistance
	Exclusively or almost exclusively with assistance

Coping with disease- or therapy-related demands		
	**Independence**	**Need for help**
Medication<br><br>Injections<br><br>Supply of intravenous access (e.g., port)<br><br>Suction and oxygen administration<br><br>Body-worn aids<br><br>Dressing changes and wound care<br><br>Stoma care<br><br>Regular intermittent catheterization and use of laxative methods	Not applicable	Daily
	Independent	Weekly

Therapeutic measures in a home environment<br><br>Time- and technology-intensive measures in a home environment<br><br>Doctor visits<br><br>Visits to other medical or therapeutic facilities (up to 3 hours)<br><br>Extended visits to other medical or therapeutic facilities (longer than 3 hours)<br><br>Adherence to a diet and other illness- or therapy-related behavioral guidelines		Monthly

**Need**	**Applies**
Doctor visits<br><br>Medications<br><br>Physical therapy<br><br>Occupational therapy<br><br>Voice, speech, language therapy<br><br>Podiatry<br><br>Special patient observation according to position 24 HKP guideline	No
	Yes

**Managing daily life and maintaining social contacts**	
Organizing the daily routine and adapting to changes	Independent

Resting and sleeping<br><br>Keeping busy<br><br>Making future-oriented plans<br><br>Interaction with people in direct contact<br><br>Maintaining contact with people outside immediate environment	Mostly independent
	Mostly dependent
	Dependent

A2. Means and probabilities based on average margins after regression models on gender, age and care grade

	Gender	Age	Care grade
C1	0.69***	77.91***	1.02***
	(0.00)	(0.10)	(0.00)
C2	0.67***	76.36***	1.01***
	(0.01)	(0.17)	(0.00)
C3	0.62***	78.49***	1.42***
	(0.00)	(0.07)	(0.00)
C4	0.60***	77.03***	2.11***
	(0.00)	(0.08)	(0.00)
C5	0.56***	81.41***	2.78***
	(0.00)	(0.06)	(0.00)
N	101208	101227	101227
